# Linkages of plant–soil feedbacks and underlying invasion mechanisms

**DOI:** 10.1093/aobpla/plv022

**Published:** 2015-03-17

**Authors:** Inderjit, James F. Cahill

**Affiliations:** 1 Department of Environmental Studies, Centre for Environmental Management of Degraded Ecosystems (CEMDE), University of Delhi, Delhi 110007, India; 2 Department of Biological Sciences, University of Alberta, Edmonton, Alberta T6G 2E9, Canada

**Keywords:** Invasion, native species, non-native ranges, soil communities, virulent pathogens

## Abstract

Soil microbial communities and processes have repeatedly been shown to impact plant community assembly and population growth. Soil-driven effects may be particularly pronounced with the introduction of plants to non-native ranges, as introduced plants are not typically accompanied by transference of local soil communities. Here we describe how the mechanisms by which soil community processes influence plant growth overlap with several known and well-described mechanisms of plant invasion. Critically, a given soil community process may either facilitate or limit invasion, depending upon local conditions and the specific mechanisms of soil processes involved. Additionally, as soil communities typically consist of species with short generation times, the net consequences of plant–soil feedbacks for invasion trajectories are likely to change over time, as ecological and evolutionary adjustments occur. Here we provide an overview of the ecological linkages of plant–soil feedbacks and underlying mechanisms of invasion.

## Abstract (precis)

Plant-soil feedbacks including soil pathogens, mycorrhizal costs/benefits and nutrient dynamics impact invasion directly or through interactions with other ecological processes. Here we describe how the mechanisms by which soil community processes influence plant growth overlap with several known and well-described mechanisms of plant invasion. We need to focus on the underlying mechanisms of soil feedbacks, and how they link to invasion along temporal scale.

## Introduction

Due to the sustained nature of plant growth, soil-mediated interactions have the potential to develop feedbacks among plants and soil communities (Klironomos 2002; Callaway *et al.* 2004; Petermann *et al.* 2008; Terborgh 2012; van der Putten *et al.* 2013). Plant–soil feedbacks (PSFs) refer to plant-induced changes in soil community structure and function that in turn impact the subsequent establishment and growth of plants (van der Putten *et al.* 2013). Common groups of organisms involved in PSFs include bacteria, arbuscular, ectomycorrhizal and pathogenic fungi, and nematodes and other soil invertebrates. Individually and combined, these organisms can in part generate plant–soil feedbacks that have neutral, positive or negative effects on plant growth (Inderjit and van der Putten 2010; van der Putten *et al.* 2013). The contingency of the net effects is a function of numerous factors both intrinsic and extrinsic to the organisms involved.

**Figure 1.**
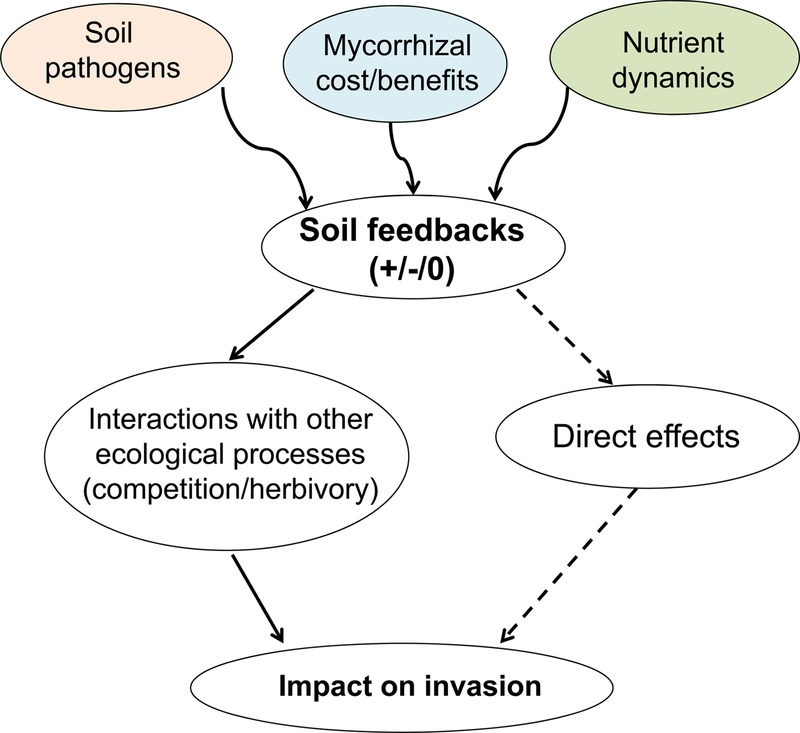
Soil feedbacks, soil pathogens, mycorrhizae and/or nutrient dynamics, impact invasion either directly or through interactions with other ecological processes including competition and herbivory.

Plant–soil feedbacks, through their impacts on plant growth, can influence a diversity of processes associated with plant invasion (Inderjit and van der Putten 2010). Most notably, PSFs are involved in enemy release, novel weapons, biotic resistance (BRH, Levine *et al.* 2004), accumulation of native soil pathogens (Mangla *et al.* 2008), shifts in litter decomposition and nutrient availability (Ehrenfeld 2003; Mata *et al.* 2013; Perkins and Nowak 2013) and disruption of mutualistic associations (Stinson *et al.* 2006; Hale *et al.* 2011; Hale and Kalisz 2012) (Table 1). Because PSFs can alter invasion mechanisms, it is not surprising that PSFs can impact the probability, speed and consequences of plant invasion in natural systems (reviews by Bever 2003; Ehrenfeld *et al.* 2005; Wolfe and Klironomos 2005; Levine *et al.* 2006; Reinhart and Callaway 2006; Inderjit and van der Putten 2010; Bever *et al.* 2012; Hodge and Fitter 2013; Suding *et al.* 2013; van der Putten *et al.* 2013; Mehrabi and Tuck 2015). Due to the intertwining of PSFs and several mechanisms of plant invasion, it is useful to discuss linkages between soil-mediated processes and plant invasion. These linkages are complex, as it becomes clear that PSFs are highly contextual. For example, soil microbial communities often exert differential impacts on a species in its native and non-native ranges (review by Inderjit and van der Putten 2010). Differences in community composition and functioning of soil communities between native and introduced soils is well known (Reinhart *et al.* 2003; Yang *et al.* 2013), though the underlying causes are uncertain and in need of more attention. When focussing on the potential linkages between PSFs and plant invasion, we suggest a useful first step would be to differentiate among PSF impacts that involve conspecific and intraspecific interactions, and those that predominantly involve interspecific and/or heterospecific feedbacks (van der Putten *et al.* 2013). Intraspecific PSFs may involve direct interactions among the soil community and the invading plants species, while interspecific PSFs will involve indirect interactions among the invading plant community, the resident plant community and the soil community (Fig. 1). We use this framework below to describe some of the ways PSFs can influence plant invasion.

**Table 1.** Different kinds of plant–soil feedbacks that could impact exotic and/or native plant species. Impacts: +, positive; −, negative; 0, neutral.

Plant–soil feedbacks	Mechanism	Impacts		Examples
		Invader	Natives	
Absence of virulent pathogens	Enemy release	+	0	Colautti *et al.* (2004)
Biotic assistance	Accumulation of native soil pathogens	0	−	Eppinga *et al.* (2006)
Biotic resistance	Pathogenesis of invader	−	0	Levine *et al.* (2004)
Mycorrhizal network disruption	Suppression of mycorrhiza	0	−	Hale and Kalisz (2012)
Impact on mutualists	Enhanced mutualists	+	0	Reinhart and Callaway (2004, 2006)
Impact on pollinators	Mycorrhizal-mediated tri-trophic interactions	0	−	Cahill *et al.* (2008)
Microbe-aided nutrient release	Soil fertility	+	0	Ehrenfeld (2003)

## Direct Plant–Soil Feedbacks

### Virulent pathogens: escape or encounter in invaded communities

A lack of virulent pathogens associated with the introduced species in the non-native range can facilitate the initial stages of invasion, an example of the enemy release hypothesis (Mitchell and Power 2003; Reinhart *et al.* 2010). Although introduced species may gain advantage in introduced habitats due to their escape from pathogens present in their home range, multiple introductions of a species increase the chances of introduction of associated pathogens (Torchin and Mitchell 2004), thereby reducing the value of ‘escape’ as a mechanism to maintain populations in a new habitat.

Not all soil-borne pathogens necessarily have the same impacts on invasion. For example, highly virulent soil-borne pathogens (e.g. *Phythium* spp., *Fusarium* spp.) that affect many plant species may more strongly limit plant growth than do specialist pathogens (Reinhart *et al.* 2010). However, relative to studies of specialist pathogens, we know less about whether generalist pathogens exert fitness pressure equally in native and non-native ranges (Parker and Gilbert 2007). Commonly, there is a lack of the data needed to characterize a microbe as ‘pathogen’, ‘generalist’ and/or ‘specialist’, particularly in the context of multiple hosts. Most ecological experiments on negative soil feedbacks do not identify the specific microbe associated with a given response, and use approaches different from what is commonly done in traditional plant pathology research.

Many studies assume that escaping specialist enemies is important but enemy release is really about escaping enemies with impact which should have more to do with virulent than avirulent enemies regardless of them being generalists, specialists or intermediates (K. Reinhart, pers. comm.; Barrett *et al*. 2009; Reinhart *et al.* 2010; Callaway *et al.* 2011). Especially since most soil-borne pathogen species known for being virulent have generalist tendencies (or at least intermediates). Enemy release emphasizes specialization and appears to assume specialists are somehow more virulent than generalists or that generalists have equal pressure in both regions (K. Reinhart, pers. comm.; Chun *et al.* 2010; Halbritter *et al.* 2012). Further effects of soil-borne pathogens vary by host species. Pathogen may be able to colonize the roots of many hosts but may be able to cause disease in only a subset. So, susceptibility may be a key issue. Filling this gap in our knowledge will be critical to understanding the relative contribution of this form of enemy release towards facilitating plant invasion. Additionally, as there may be different evolutionary responses to specialist and generalist pathogens (Jarosz and Davelos 1995; Parker and Gilbert 2004), the specificity of the pathogen to the host may influence the long-term stability of PSF-mediated mechanism of plant invasion.

## Indirect Plant–Soil Feedbacks

### Soil pathogen-mediated biotic resistance

Direct interactions between invaders and soil pathogens can be driven by the activities of the resident plant community, not the invader itself. For example, native species may culture pathogens that can also infect the invader, an example of soil-mediated biotic resistance (Maron and Vilà 2001; Levine *et al*. 2004; Nijjer *et al*. 2007; Halbritter *et al.* 2012; Flory and Clay 2013). For example, European beach dune grass *Ammophila arenaria* experiences both enemy release and biotic resistance in non-native ranges, and it is the balance between these mechanisms that determines its invasiveness (Knevel *et al.* 2004).

### Soil pathogen-mediated biotic assistance

Some invaders cultivate and accumulate local soil pathogens that inhibit native species more than themselves, a form of apparent competition (Eppinga *et al.* 2006; Mangla *et al.* 2008). When invaders enhance the growth of pathogens of the native plants, the PSF provides a form of biotic assistance to the introduced species. In some situations, pathogens hosted by the invader could suppress the establishment and growth of native species, the phenomenon is identified as ‘spillover’ by Flory and Clay (2013). This is directly analgous to the previously discussed biotic resistance that PSFs may also provide in some invasion scenarios.

### Positive impacts on soil mutualists

Mutualists may have more positive impacts in non-native ranges than the native ranges of the invaders, as predicted by the enhanced mutualists hypothesis (EMH) (Reinhart and Callaway 2004, 2006; Sun and He 2010). For example, the neutral to negative impacts of PSFs on invaders such as *Triadiaca sebifera* (Chinese tallow) in its native range China compared with positive PSFs in its non-native range USA was linked to the higher levels of AMF colonization and greater net benefits to the invader in USA than in China (Yang *et al.* 2013), which supports EMH. However, invasion may not always be linked to EMH. For example, Callaway *et al.* (2011) studied the effect of soil biota from native and expanded ranges in USA, and invasive European ranges of *Robinia pseudoacacia*. These authors did not find any role of mutualistic N-fixing organisms in the invasion of *R. pseudoacacia*. The various components of soil communities therefore may have differential impacts on the invader, which make evolutionary relationships and spatial soil heterogeneity important.

### Negative impacts on soil mutualists

In some situations, soil mutualists may not directly impact an invader, but instead a decline in soil mutualists may reduce the performance of native hosts, thereby indirectly benefitting the invader. For example, some invasive species can chemically suppress AMF, disrupting mutualistic associations among local tree seedlings and mycorrhizal fungi and suppressing the establishment and growth of local trees (Stinson *et al.* 2006; Hale and Kalisz 2012). Meinhardt and Gehring (2012) reported a non-mycotrophic invasive tamarisk (*Tamarix* sp.) suppressed native *Populus fremontii* by disrupting its mutualistic associations between AMF and ectomycorrhizal fungi and *P. fremontii*.

### Soil community mediated impacts on resource availability

In addition to the impacts of pathogens and mutualists described above, soil microbial communities can influence soil fertility through decomposition of litter, which in turn may influence invasion. Any difference in the chemical composition of exotic and native litter (e.g. higher C : N ratios in litter) could cause a shift in the decomposition rate of the litter (Hawkes *et al.* 2005; Rout and Chrzanowski 2009; van der Putten *et al*. 2013), thereby altering soil fertility and invasion trajectories. These plant-induced soil processes develop feedbacks between and among plants and soil communities, which may facilitate the establishment and growth of an invader (Bajpai and Inderjit 2013).

Soil microbial communities may be an important driver of litter-nitrogen release, facilitating invasion. Soil invaded by an aggressive invader, *Ageratina adenophora*, had higher values for available nitrogen and microbial respiration compared with soils not yet invaded (Bajpai and Inderjit 2013). Terpene-rich litter of *A. adenophora* was linked to the higher soil microbial activity that results in the release of nitrogen from decomposing litter. Litter-released nitrogen facilitates the growth of *A. adenophora*. Although soil pathogens are largely responsible for die-off in its own soil in non-native ranges. The higher biomass accumulation of *Bromus tectorum* (cheatgrass) in its die-off monoculture stands was probably due to higher nitrate-nitrogen (Meyer *et al.* 2014).

Microbial-aided decomposition of exotic litter of some invaders may lead to higher release of nutrients upon compared with native litter as a consequence of greater organic C in soil (Ehrenfeld *et al.* 2005; Hata *et al*. 2012; Kaur *et al.* 2012; Meisner *et al*. 2012). Although several studies have investigated the interactions among invasion, soil communities and decomposition, the focus is typically on inorganic nitrogen. Many plant species are also able to use organic forms of nitrogen, both directly and indirectly (Näsholm *et al.* 2009), and its role in plant invasion may have been overlooked. The root foraging behaviour (*sensu* Cahill and McNickle 2011) of most species, invasive and otherwise, is poorly understood.

## Contingencies in PSF in Temporal and Evolutionary Contexts

**Figure 2.**
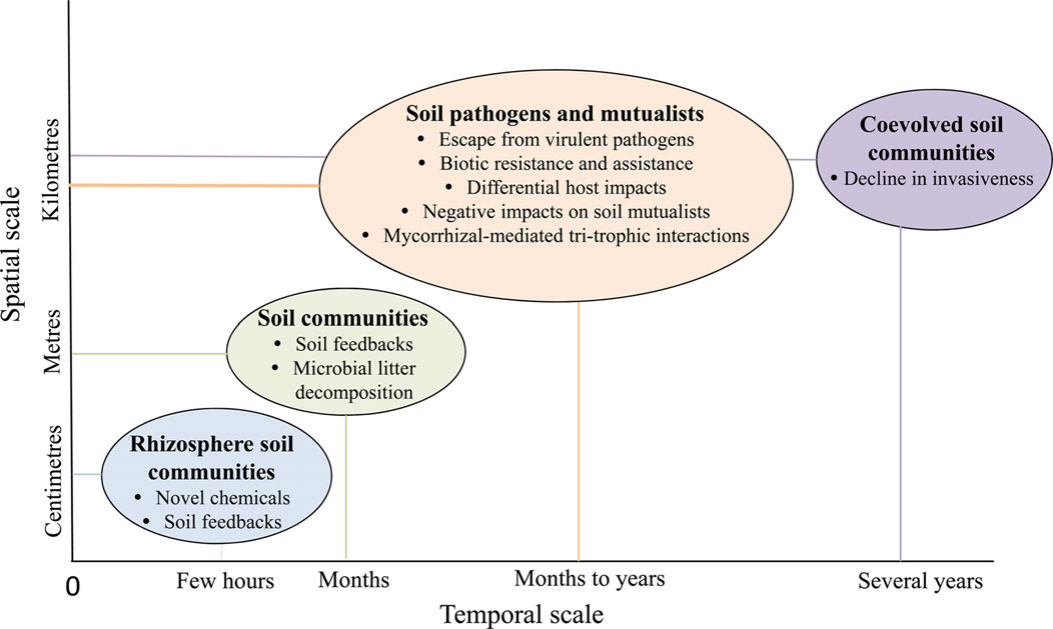
Interaction of soil communities within introduced ranges along spatial and temporal scales. The spatial variation in the soil microbial communities could impact ecosystem components and processes on spatial and temporal scales (Kardol *et al.* 2006). Root exudates of the exotic and native species can manipulate plant–soil feedbacks in the rhizosphere (Lange *et al.* 2014). Rhizosphere soil communities could impact accumulation and functioning of chemicals in native and invaded ranges at very small spatial and temporal scales (Inderjit *et al*. 2006, 2011). Plant species may experience negative soil feedbacks in its native range, and neutral to positive impact in its non-native ranges at small spatial and temporal (metres and months, respectively) scales (Callaway *et al.* 2004). Plant–soil feedbacks from heterospecific vs. conspecifics could impact a species in small scales. At larger spatial (hundreds to thousands of kilometres) and temporal (months to years) scales, escape of an invader from virulent pathogens, native soil pathogens and accumulation of native soil pathogens facilitate invasion. Soil mutualists may impact invasion by mediated tri-trophic interactions and manipulating soil fertility. In addition any negative impact of invader on soil mutualists may facilitate invasion. At still larger temporal (decades to hundreds of years), the invader may experience new enemies, resistant neighbours, coevolved soil communities that can result in establishment of native plant species and naturalization of other exotic plant species. Reproduced after modification with permission from the publisher (Inderjit *et al.* 2011).

The introduction of a novel plant species to a community can cause compositional and functional shifts in soil communities, which can lead to shifts over time in PSFs and their impact in the invasion process. As invasion is an ongoing process, with different communities within an invaded region varying in time since invasion, there may be substantial temporal and spatial complexity in the net effects of PSFs and their influence on invasion (Fig. 2). The overarching goal of this paper is to develop linkages between soil microbial communities-mediated processes and invasion, and to discuss contingencies in PSFs in temporal and spatial contexts, in a unique manner not addressed previously.

## Time Since Invasion

Over time, the introduced species is no longer ‘novel’, and the local microbial populations will likely have responses through population and evolutionary changes, with dynamics eventually stabilizing. Under stable-state conditions, there is no expectation that PSF-mediated invasion mechanisms that operated during the transient dynamics soon after introduction will continue in the same direction or intensity. Thus, we suggest PSFs may have differential roles in the processes of invasion and persistence (Hawkes 2007).

One of the assumptions of the novel weapons hypothesis (Callaway and Ridenour 2004) is that naïve soil communities in the non-native ranges could not use novel chemicals, and thus indirectly helps in building phytotoxic pool of novel chemicals (Callaway and Ridenour 2004). Interacting species within soil communities may coevolve over time, increasing resistance to the invader with time since invasion (Lankau 2010). For examples, coevolved/resistant soil communities in the late invasion stages may able to break down allelochemicals released by the invader, while early-stage soil communities may not have this functional ability. This idea is supported by Lankau (2011*a*), who found that microbial richness near *Alliaria petiolata* declined with age of invasion, and native community become more resistance to *A. petiolata* invasion with increasing age of invasion. The community's resistance to *A. petiolata* invasion resulted in the (i) establishment of sensitive soil microbes and (ii) increase in the abundance of native woody species (Lankau *et al.* 2009; Lankau 2011*a*, *b*). Information on invasion age of sites would help to understand any variation in the impact of soil communities in invasion process (see also Strayer *et al.* 2006).

The net effect of soil pathogens-mediated feedbacks on plant invasion will be a function of degree of their abundance and their virulence. As is common to many host–pathogen systems, the intensity of interaction can change over time, due to a diversity of ecological and evolutionary responses of the interacting species (Tack and Laine 2014). We need to understand the mechanisms of long-term interactions in native vs. non-native ranges to understand whether they would be stable or labile over time.

## Spatial Heterogeneity

Positive or negative PSFs in the non-native ranges do not necessarily occur in all locations largely because ecosystem processes and habitat factors may influence the underlying mechanisms driving PSFs. Significant variation in the strength of negative PSFs in native European soils (Maron *et al.* 2014) suggests the need to identify soil pathogens and to carry out more biogeographic studies to quantify the PSFs on different species. The pathogenic effects may be irregularly distributed over the range of a species and vary across native vs. non-native ranges. The distribution of the plant and pathogen may also be temporally variable. It may be that an effect for a given location is entirely unique relative to the majority of the rest of the species’ range. Therefore, heterogeneity and temporal flux of soil communities are important to learn that PSF are spatially and temporally variable.

An entirely different approach could be taken to avoid the confounding effects of different soil types/textures of soil inoculum from across ranges or among sites per range. Approaches such as pathogenicity or mycorrhizal dependency enable testing effects of specific microbes from different regions in a standard growth media to determine shifts in interaction strength (e.g. Reinhart *et al.* 2010). This helps to lessen the black box of describing general soil biota effects and numerates effects of specific soil component parts.

## Summary

The mechanisms underlying PSFs, including microbial population growth, plant competition and mutualistic benefits, are all contingent upon local conditions such as herbivory (Bezemer *et al.* 2013), soil texture (Schradin and Cipollini 2012), soil fertility and heterogeneity (Heather and Haubensak 2008), plant species and soil conditioning (Harrison and Bardgett 2010), root exudates (Lange *et al.* 2014) and soil community density (Aguilera 2011). Any variation in the mechanisms underlying PSFs along gradients or with time would impact the net PSF observed (Ehrenfeld *et al.* 2005). We have a lack of empirical evidence on the biogeographic comparisons of the direct effect of mycorrhizae on invader's growth in its native vs. non-native ranges. Such effects would likely depend on interactions with soil fertility (N : P ratios) and specific mycorrhizal associations from native and non-native ranges. Interactions among mechanisms further enhance the biocomplexity of the system, and decrease generality across space and time. It is not practical to design individual field studies to include each and every potential contributing factor, nor is it likely effective to continue to (typically) ignore the role site variation can play in determining PSFs and their impacts on invasion trajectories. However, a current lack of capacity to manipulate and study this complexity does not reduce its potential importance to the processes governing species invasion.

Future work on the interaction of PSFs with other invasion mechanisms in field situations would help to understand PSFs at bigger spatial and temporal scales, although there are obviously legal and ethical issues associated with large field experiments in invasive species. The role of PSFs in invasion should be evaluated in examining PSFs interactions with other ecological components such as herbivores, competitors, consumers, chemicals, abiotic factors and habitat heterogeneity. Further research on the adding trophic interactions and their spatial complexity on top in terms of biogeographic comparisons of the impacts of mycorrhizal fungi on plant defense against enemies, pollinators, competitors, would be interesting but likely not compelling or even logistically not feasible.

There is a need for taking the complexities of plant–soil feedback impacts on invasion into account to better understand when soil communities are, or are not, critical contributors towards the facilitation and resistance of plant invasion.

## Sources of Funding

The Council of Scientific & Industrial Research (CSIR) supported Inderjit's work and Natural Sciences and Engineering Research Council (NSERC) supported the research of J.F.C.

## Contributions by the Authors

Both authors contributed equally.

## Conflict of Interest Statement

None declared.
